# *Streptococcus pneumoniae* Cell Wall-Localized Trigger Factor Elicits a Protective Immune Response and Contributes to Bacterial Adhesion to the Host

**DOI:** 10.1038/s41598-019-40779-0

**Published:** 2019-03-12

**Authors:** Aviad Cohen, Shani Troib, Shahar Dotan, Hastyar Najmuldeen, Hasan Yesilkaya, Tatyana Kushnir, Marilou Shagan, Maxim Portnoi, Hannie Nachmani, Rachel Benisty, Michael Tal, Ronald Ellis, Vered Chalifa-Caspi, Ron Dagan, Yaffa Mizrachi Nebenzahl

**Affiliations:** 1 The Shraga Segal Department of Microbiology, Immunology and Genetics, Faculty of Health Sciences, Ben-Gurion University of the Negev, Beer-Sheva, Israel; 2 NasVax Ltd., Ness Ziona, Israel; 3 Department of Infection, Immunity and Inflammation to Department of Respiratory Sciences, University of Leicester, Leicester, United Kingdom; 4 Department of Biology, College of Science, University of Sulaimani, Sulaimani, Iraq; 5 Bioinformatics Core Facility, National Institute for Biotechnology in the Negev (NIBN), Ben-Gurion University of the Negev, Beer-Sheva, Israel; 6 The Faculty of Health Sciences, Ben-Gurion University of the Negev, Beer-Sheva, Israel

## Abstract

Trigger factor (TF) has a known cytoplasmic function as a chaperone. In a previous study we showed that pneumococcal TF is also cell-wall localized and this finding combined with the immunogenic characteristic of TF, has led us to determine the vaccine potential of TF and decipher its involvement in pneumococcal pathogenesis. Bioinformatic analysis revealed that TF is conserved among pneumococci and has no human homologue. Immunization of mice with recombinant (r)TF elicited a protective immune response against a pneumococcal challenge, suggesting that TF contributes to pneumococcal pathogenesis. Indeed, rTF and an anti-rTF antiserum inhibited bacterial adhesion to human lung derived epithelial cells, indicating that TF contributes to the bacterial adhesion to the host. Moreover, bacteria lacking TF demonstrated reduced adhesion, *in vitro*, to lung-derived epithelial cells, neural cells and glial cells. The reduced adhesion could be restored by chromosomal complementation. Furthermore, bacteria lacking TF demonstrated significantly reduced virulence in a mouse model. Taken together, the ability of rTF to elicit a protective immune response, involvement of TF in bacterial adhesion, conservation of the protein among pneumococcal strains and the lack of human homologue, all suggest that rTF can be considered as a future candidate vaccine with a much broader coverage as compared to the currently available pneumococcal vaccines.

## Introduction

The commensal bacterium *Streptococcus pneumoniae* continues to cause morbidity and mortality worldwide^1^. Since the implementation of pneumococcal capsular polysaccharide vaccines^2,3^ a substantial reduction in disease burden has been reported. While the 23 valent unconjugated pneumococcal polysaccharide vaccine (PPSV23) was found to be 45–65% effective in immunocompetent adult patients^2^, this vaccine, unfortunately, does not elicit an immune response in the group with the highest rate of pneumococcal disease burden, i.e., children younger than two years of age^4,5^. The first commercial version of the pneumococcal conjugate vaccine (PCV), which included 5 capsular serotypes, has evolved over the last three decades to include up to 15 capsular polysaccharide serotypes^6,7^. PCVs induce immune memory and a protective immune response in infants, but only protect against serotypes that are included in the vaccine^8,9^. Limitations of the currently available polysaccharide vaccines and the continuous increase in antibiotic resistance to *S. pneumoniae* underscore the urgency of the need for pneumococcal vaccines with broader coverage^9–12^.

Toward the development of protein-based vaccines, candidate pneumococcal immunogenic surface proteins are identified by proteomics- and bioinformatics-based analyses^13–17^. The identified proteins are screened for low or no homology to human proteins and then tested for their vaccine potential. Using this working scheme several immunogenic proteins have been identified and shown to elicit protective immune responses in mouse models. Among these proteins are: pneumococcal surface protein A (PspA)^18^, the histidine triad motif (Pht) A, B, D and E proteins^19^, fructose bisphosphate aldolase (FBA)^20^, glutamyl tRNA synthetase (GtS)^21^, pneumococcal serine-rich repeat protein (PsrP)^22^, PcsB and StkP^23^ and nucleoside ABC transporter component)^24^. The presumed function of these proteins in pneumococcal pathogenicity and physiology were then studied further. For example, PhtD controls Zinc homeostasis in the bacterium^25^ and GtS, PsrP and FBA were found to function as adhesins^21,22,26^.

We have previously determined that the *S. pneumoniae* trigger factor (TF) in addition to its known cytoplasmic function, is immunogenic in mice and that it is cell-wall (CW) localized^27,28^. TF is a heat shock protein that binds the ribosome in the cytoplasm with its N terminal domain and encounters, co-translationally, the nascent protein chain emerging from the ribosome by its C terminal chaperone domain. This function protects the newly synthesized protein from degradation and assists in its proper folding and maturation^29^. In addition, the central part of TF catalyzes peptidyl-prolyl cis-trans isomerization, which further contributs to the proper folding of proteins^30^. TF can be found in all eubacteria, with variable degrees of homology, but not in yeast or mammalian cells^30^. In *S. pyogenes* TF belongs to the highly conserved anchorless CW proteins with no cross reactivity to human proteins^31,32^. The intentation of the current study was to determiner whether *S. pneumoniae*-derived TF is capable of eliciting protective immune responses in a mouse model of *S. pneumoniae* infection and reveal the function of the CW-localized TF in pathogenesis.

## Results

### Bioinformatic analysis of trigger factor

#### Characterization of Trigger factor

TF is a unique bacterial protein with no homologue in the human genome. Blast analysis demonstrated that TF is highly conserved (98% identity) among the available *S. pneumoniae* sequenced strains in the NCBI database. BlastP of *S. pneumoniae* TF against other bacteria identified a TF protein with 96% homology in *S. mitis*, 76% in *S. pyogenes*, 66–76% in *S. agalactiae* and 38% in *Clostridium difficile*. Notably, the homology to several strains of *E. coli* varied between 31–76%.

The *S. pneumoniae* TF protein and its *Streptococcus* orthologs produced an un-gapped multiple sequence alignment, except for an “extruding” sequence in the N terminus of one of the proteins, which was removed from the alignment. A histogram of the conservation score per position, calculated by Jalview, was added below the alignment. B-cell linear epitopes were predicted *in silico* by four different prediction programs, however there was only partial agreement between the programs. Epitopes were found both in highly conserved and in less conserved regions of the alignment (Supplementary Fig. S1). We cannot conclude whether putative antigenicity regions of the *S. pneumoniae* TF are preferably located in *S. pneumonia* conserved or in unique regions. Thus, further studies are necessary to determine whether an immunization with TF may affect other *Streptococcus* species which may be present in the natural microbiota.

### Recombinant (r)TF characteristics

**Figure 1:**
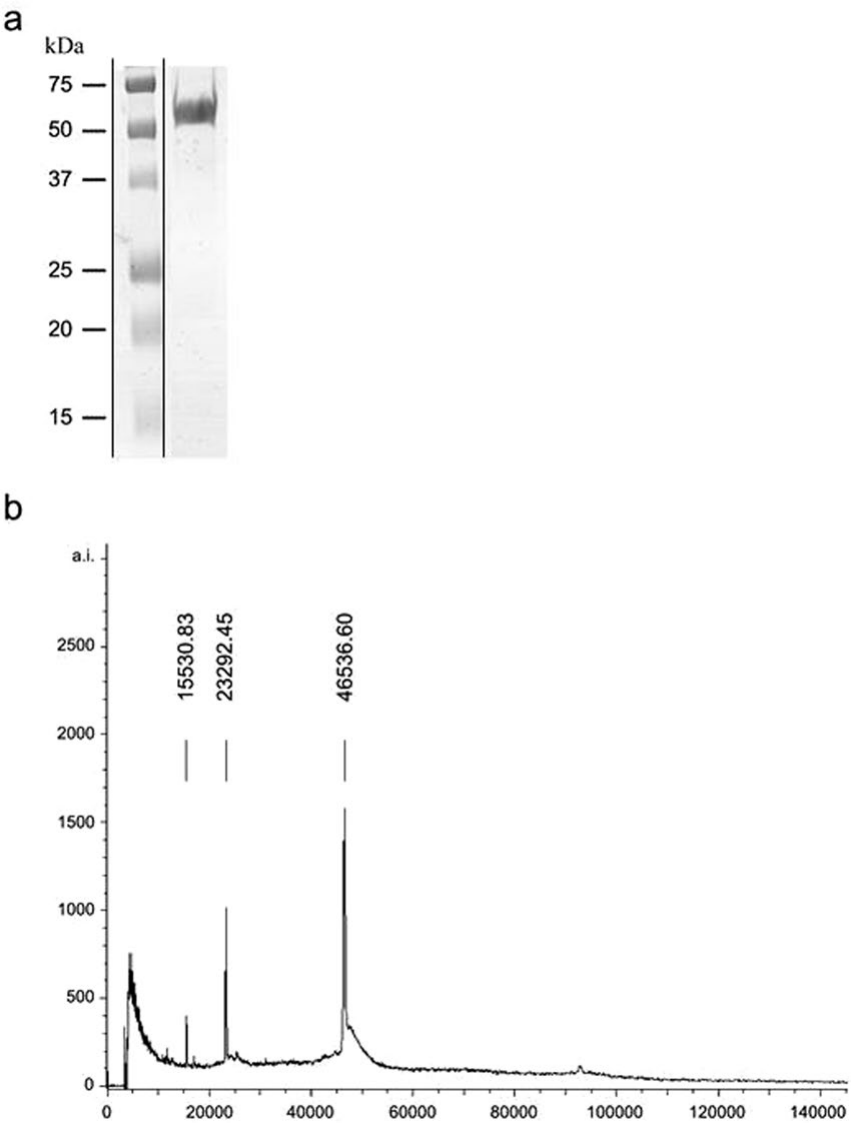
rTF characteristics. Commercially produced and purified rTF was analyzed on SDS-PAGE. A band corresponding to 55 kDa was identified by (**a**) Coomassie brilliant blue staining and (**b)** Mass spectroscopy analysis.

A custom-designed and commercially produced rTF was separated on SDS-PAGE and a band corresponding to 55 kDa was observed (Fig. 1a). The discrepancies between the expected (47190 Da) and the observed molecular weight may result from the altered electrophoretic mobility of rTF. A mass spectrometry (MS) analysis (Fig. 1b) identified a major peak with molecular mass of 46536 Da, which is different from the theoretical molecular weight of 47190 Da and this discrepancy may result from a N or C terminal truncation of the protein. MS analysis of the major peak peptide composition had 73% sequence coverage of trigger factor from *S. pneumoniae* TIGR4 strain (gi: 15900319, queried on October, 2009).

### Cell wall localization of TF

**Figure 2:**
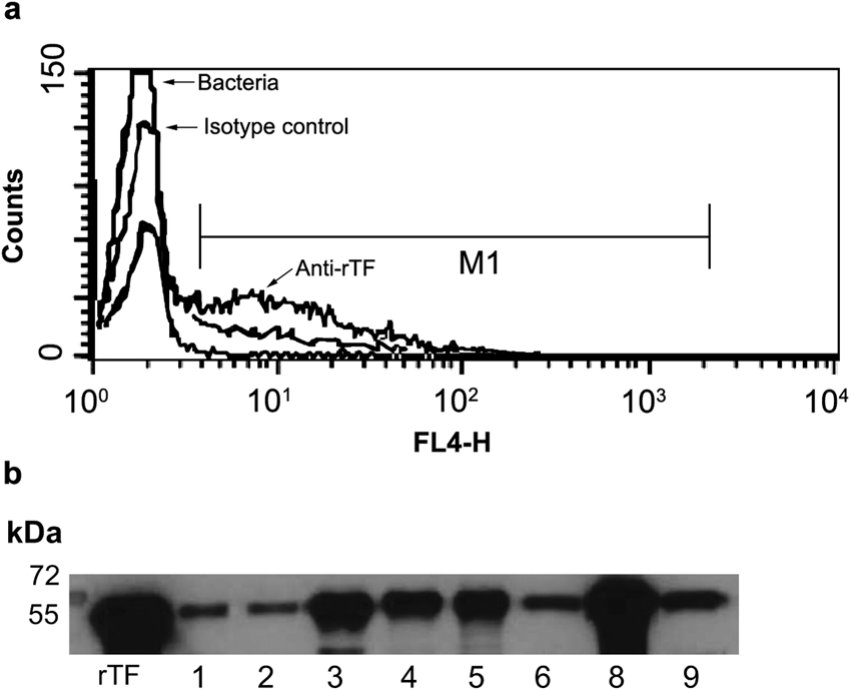
CW localization of TF. (**a**) R6 bacteria were incubated with either: i. anti-rTF mAb or ii. isotype control mouse serum (as indicated) or iii. phosppate buffered seline. All were stained with Alexa Fluor 647^®^-conjugated goat-anti-mouse-IgG as a secondary antibody and then analyzed by flow cytometry. (**b**) Thirty micrograms of protein of CW fractions from 60*S. pneumoniae* clinical strains (see Supplementary Table S2) were loaded per lane, subjected to SDS-PAGE, and immunoblotted. Purified untagged rTF (0.01 μg) was loaded as a positive control. A representative blot is shown cropped from the 15 min exposed blot is presented.

Previously we found TF in the CW fraction of *S. pneumoniae*^27,28^. Notably, we recently showed that a *bona fide* cytoplasmic protein, malonyl-CoA:ACP transacylase (FabD), which is involved in lipid metabolism could not be found in the CW protein extract^28,33^. To further verify that TF is indeed CW-localized, a monoclonal anti-rTF antibody was incubated with a live unencapsulated, serotype 2-derived, R6 strain and flow cytometry analysis was performed. TF was detected on the surface of approximately 50% of the bacteria that had been tested, confirming the surface localization of TF (Fig. 2a). Of note, antibodies cannot penetrate the cytoplasmic membrane to access the cytoplasm-localized TF and thus can detect only the CW-localized TF.

A CW protein fraction from 60*S. pneumoniae* clinical strains (Supplementary Table S2) was extracted and separated by SDS-PAGE. Immunoblotting was performed with a rabbit anti-rTF antiserum. If TF band was not detected, increased loading or exposure time were performed and resulted in positive signal (Supplementary Fig. S2). All tested strains demonstrated a band corresponding to the molecular weight of untagged rTF (Fig. 2b, Supplementary Table S2).

### TF immunogenicity

In a previous study we showed that TF is immunogenic in mice^27^. To determine the extent of antibody production following immunization with rHis-TF, ELISA assays were performed. Sera that were obtained following the second and third immunizations (TF II and TF III, respectively) were analyzed on rHis-TF coated plates. The antibody titer in both TF II and TF III was found to be 1:121,500 (see Supplemetary Fig. S3a), demonstrating the ability of rTF to elicit a substantial immune response. TF II and TF III antibody responses were significantly higher than those observed with serum obtained from mice that were immunized with another pneumococcal recombinant His tagged protein, namely rPtsA (PtsA III; See Supplementary Fig. S3a; one way ANOVA with the Dunnett post-hoc test, p = 0.0014 and p = 0.0008, respectively). PtsA III, (but not TF II and TF III), successfully reacted with rHis-PtsA coated plates (see Supplementary Fig. S3b; one way ANOVA, with the Dunnett post-hoc test, p = 0.0001). Taken together, these results establish the immunogenicity of TF and the specificity of the anti-rTF antiserum.

### rTF elicits a protective immune response in mice

**Figure 3:**
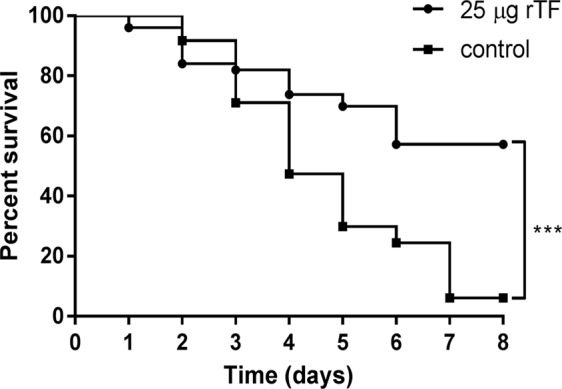
rTF elicits a protective immune response in mice. BALB/c mice were immunized with rTF emulsified with CFA and subsequently boosted with rTF emulsified in IFA. Control mice were immunized with the adjuvant only. Two weeks following the final immunization, mice were challenged intranasally with a lethal dose (1 × 10^8^ CFU) of WU2 and survival was monitored daily. The figure is a summary of three independent experiments. The extent of survival was determined by using the Log-rank (Mantel-Cox) test (n = 45 in each group; ***p = 0.0005).

The findings that TF is (a) immunogenic, (b) lacks homology to human proteins, (c) is CW localized and (d) conserved within *S. pneumoniae* strains, prompted us to test the ability of rTF to elicit a protective immune response. Immunizing BALB/c mice with rHis-TF significantly reduced mortality following a lethal intranasal challenge with the WU2 wild type (WT) strain, as compared to immunization with the adjuvant only [Fig. 3; n = 45 in each group, Log Rank (Mantel-Cox) test, p = 0.0005]. These findings highlight the vaccine potential of rTF.

### Cell wall-localized TF contributes to *S. pneumoniae* adhesion to the host

**Figure 4:**
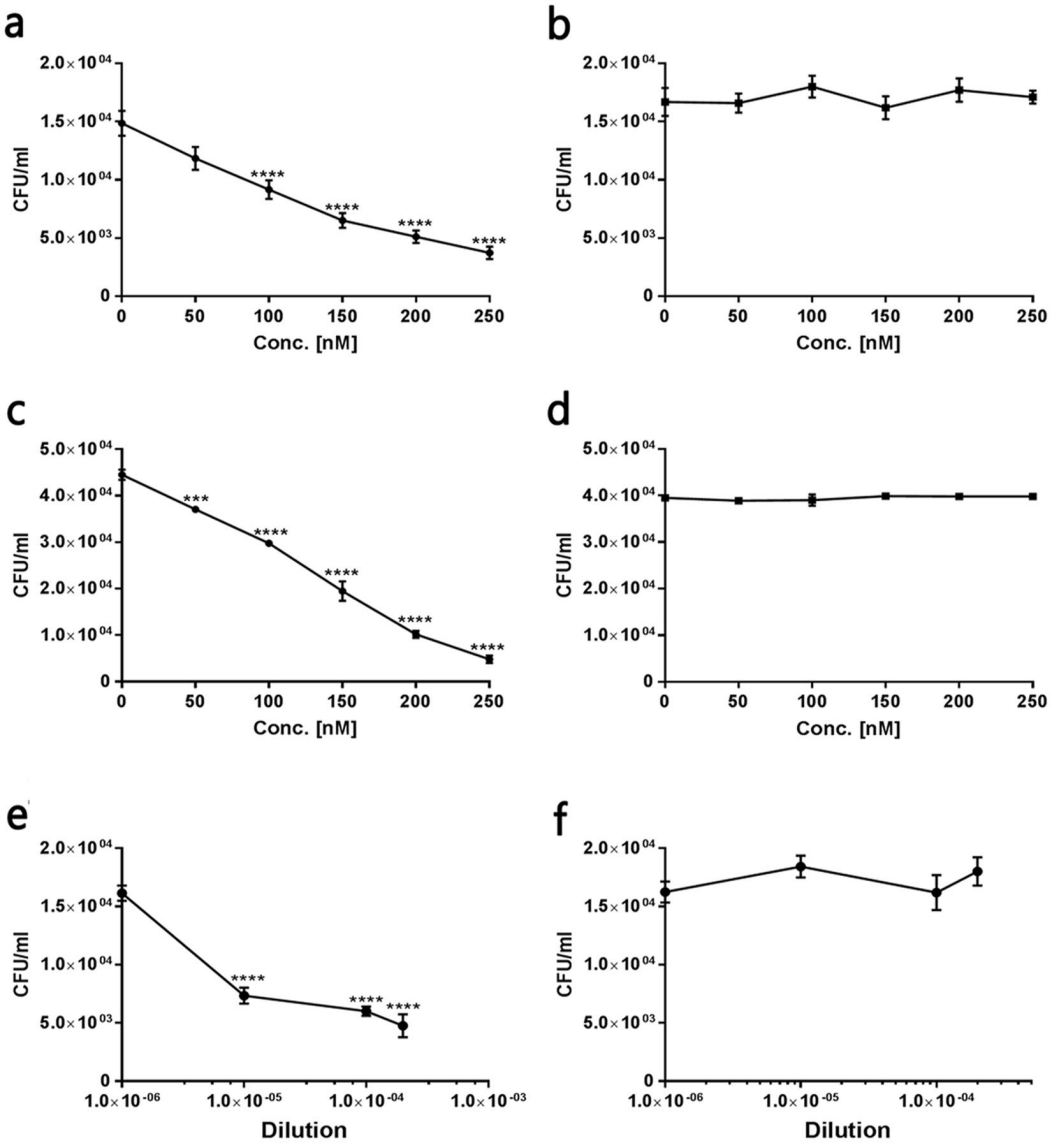
Cell wall-localized TF contributes to the adhesion of *S. pneumoniae* to the host. A549 cells were cultured in 96-well plates. Twenty four hours later, the cells. (~10^5^ cells/well) were incubated for 1 h with rHis-TRX-TF (0–500 nM). Excess protein was removed and *S. pneumoniae* were added at a MOI of ~100:1 for an additional 1 h incubation. Unattached bacteria were removed and cells were released with trypsin and plated onto blood agar plates for bacterial enumeration. rHis-Trx was used as a negative control. Values are the mean of three independent experiments, conducted in triplicates. (**a**) Extent of WU2 adhesion in the presence of rHis-Trx-TF (Spearman’s correlation p = 0.0027; r = −1; one-way ANOVA with the Dunnett post-hoc test, ****p < 0.0001 for 100–250 nM). (**b)** Extent of WU2 adhesion in the presence of r-His-Trx. (**c**) Extent of R6 adhesion in the presence of rHis-Trx-TF (Spearman’s correlation p = 0.0027; r = −1; one way ANOVA, Dunnett post-hoc test 50 nM: ***p = 0.0001; 100–250 nM: ****p < 0.0001). (**d**) Extent of R6 adhesion in the presence of rHis-Trx. (**e**) Extent of WU2 adhesion in the presence of anti rHis-Trx (Spearman’s correlation r = −1, p = 0.083; one-way ANOVA with the Dunnett post-hoc test, ****p < 0.0001). (**f)** Extent of WU2 adhesion in the presence of pre-immune serum.

The ability of rTF to elicit a protective immune response suggests that TF contributes to bacterial virulence. Many cell wall-localized proteins were found to be involved as adhesins in *S. pneumoniae* interaction with the host^21,22,26,34^. Thus we tested the ability of rHis-Trx-TF to interfere in bacterial adhesion to cultured human lung derived epithelial cells (A549 cells)^35–37^. Indeed, rHis-Trx-TF significantly inhibited the adhesion of WU2 WT to the cells in a dose dependent manner (Fig. 4a; Spearman’s correlation p = 0.0027; r = −1). Substantial reduction relative to the adhesion in the absence of rHis-Trx-TF was observed upon the addition of as little as 50 nM of rHis-Trx-TF and this reduction became statistically significant at 100 nM or higher concentrations (Fig. 4a; one way ANOVA, with the Dunnett post-hoc test p < 0.0001). Purified rHis-Trx served as the negative control and, indeed, did not interfere in the adhesion of WU2 WT to A549 cells (Fig. 4b). Similar results were obtained with the His-rTF protein (data not shown) but, we present here only the results of rHis-Trx-TF, which provided us with an internal negative control as the expressed and purified rHis-rTrx protein that excluded the possibility that either rHis or rTrx are involved in adhesion.

Our analysis also revealed that rHis-Trx-TF significantly inhibited, in a dose dependent manner, the adhesion of the unencapsulated R6 strain to A549 cells, whereas rHis-Trx had no effect on the adhesion (Fig. 4c; Spearman’s correlation p = 0.0027; r = −1, one-way ANOVA with the Dunnett post-hoc test: p < 0.0001, Fig. 4d, respectivly). A significant reduction in adhesion, relative to adhesion in the absence of rHis-Trx-TF, was observed in all tested concentrations (Fig. 4c; one way ANOVA, Dunnett post-hoc test 50 nM: p = 0.0001; 100–250 nM: p < 0.0001). Next we tested the ability of sera obtained from rHis-TF immunized mice to inhibit the adhesion of *S. pneumoniae* to A549 cells. A post-immune serum significantly inhibited WU2 WT adhesion to A549 cells in a dose-dependent manner but a pre-immune serum did not inhibit the adhesion of the bacteria to the cells (Fig. 4e; Spearman’s correlation r = −1, p = 0.083; one way ANOVA, with the Dunnett post-hoc test p < 0.0001, Fig. 4f, respectively). Taken together, these results demonstrate the contribution of CW-localized TF to the adhesion of *S. pneumoniae* to host cells.

### Lack of TF reduces *S. pneumoniae* adhesion to host cells

**Figure 5:**
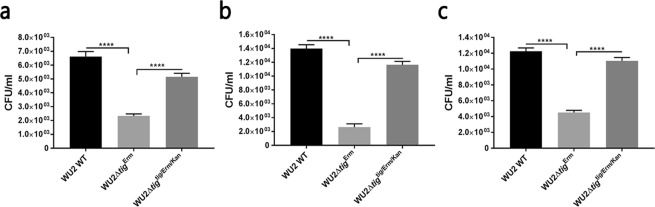
The lack of TF reduces the adhesion of *S. pneumoniae* to host cells. (**a**) The extent of adhesion of WU2Δ*tig*^Erm^ compared to that of the WU2 WT strain and the complemented strain WU2Δ*tig*^*tig*/Erm/Kan^ to A549 cells (one-way ANOVA with the Dunnett post-hoc test, ****p < 0.0001). (**b**) The extent of adhesion of the WU2Δ*tig*^Erm^ strain, compared to that of the WU2 WT and WU2Δ*tig*^*tig*/Erm/Kan^ strains, to NSC 34 cells (one-way ANOVA with the Dunnett post-hoc test, ****p < 0.0001). (**c**) The extent of adhesion of the WU2Δ*tig*^Erm^ strain, compared to that of the WU2 WT and to WU2Δ*tig*^*tig*/Erm/Kan^ strains, to U251 cells (one-way ANOVA with the Dunnett post-hoc test, ****p < 0.0001).

To confirm that TF contributes to adhesion of *S. pneumoniae* to the host, we created a mutant that lacks TF (WU2Δ*tig*^Erm^) and a *tig*-complemented strain (WU2Δ*tig*^*tig*/Erm/Kan^). We tested the extent of the adhesion of these strains to A549 cells^35–37^, to the murine motor neuron NSC 34^38^ cells and to the human glioblastoma cells U251 cells^39^. The extent of adhesion of WU2Δ*tig*^Erm^ to A549 cells was significantly reduced compared to both WU2 WT and the complemented strain WU2Δ*tig*^*tig*/Erm/Kan^ (Fig. 5a; one way ANOVA, with the Dunnett post-hoc test p < 0.0001), suggesting that TF contributes to *S. pneumoniae* adhesion to host cells. A similarly reduced adhesion to A549 cells was observed with an additional *tig* mutant WU2Δ*tig*^kan^ compared to WU2 WT (data not shown). The finding that WU2Δ*tig*^*tig*/Erm/Kan^ regained most of its capacity to adhere to the A549 cells indicates that the reduced adhesion observed in WU2Δ*tig*^Erm^ is not the result of a polar effect. To exclude the possibility that differences in growth rates accounted for the altered extent of adhesion we compared the growth rates of WU2Δ*tig*^Erm^, WU2 WT and WU2Δ*tig*^*tig*/Erm/Kan^ under anaerobic conditions and found them to be similar during the midlog stage, with estimated doubling times of 60, 78 and 72 minutes respectively, differences deemed insignificant. Following the midlog stage the growth of mutant strain was slightly attenuated in comparison to the wild type and complemented strains, reaching final OD_600_ of only 0.758, as opposed to final OD_600_ of 0.807 and 0.798 recorded for wild type and complemented strain, respectively (see Supplementary Fig. S4). Notably the growth rate of WU2Δ*tig*^kan^ mutant was similar to that of the WU2 WT WU2, in both aerobic (74.8 minutes and 69.8 minutes, respectively) and anaerobic (65 minutes and 61 minutes, respectively) conditions, with no significant variances. Significantly, we found that the mutated WU2Δ*tig*^Erm^, and the complemented strain WU2Δ*tig*^*tig*/Erm/Kan^ were identical to the WU2 WT strain in terms of colony morphology (data not shown).

The extent of adhesion of WU2Δ*tig*^Erm^ to NSC 34 cells was also significantly reduced as compared to that of WU2 WT and WU2Δ*tig*^*tig*/Erm/Kan^ (Fig. 5b; one way ANOVA, with the Dunnett post-hoc test p < 0.0001). The extent of adhesion of WU2Δ*tig*^Erm^ to U251 cells was significantly lower compared to WU2 WT and WU2Δ*tig*^*tig*/Erm/Kan^ (Fig. 5c; one way ANOVA, with the Dunnett post-hoc test p < 0.0001).

### TF contributes to *S. pneumoniae* virulence *in vivo*

**Figure 6:**
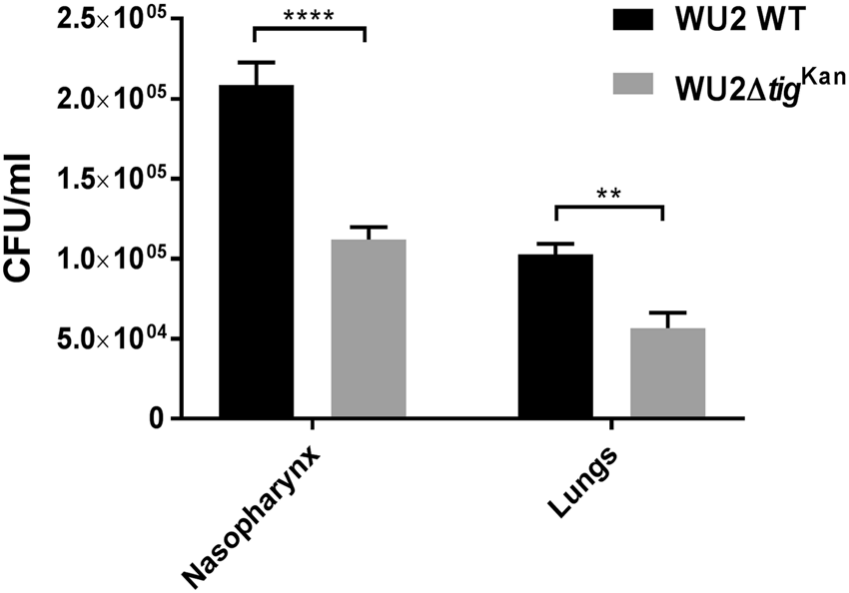
TF contributes to *S. pneumoniae* virulence *in vivo*. BALB/c mice were inoculated intranasally with a sublethal dose (5 × 10^7^ CFU) of the WU2 WT or WU2Δ*tig*^kan^ strains. Forty-eight hours following the inoculation, the mice were euthanized and their nasopharynx and lungs were excised, homogenized, and plated onto blood agar plates for enumeration. Results are a summary of three independent experiments (n = 15 in each group; Student *t*-test, ****p = 0.0001 and **p = 0.0031, respectively).

To assess the involvement of TF in virulence *in vivo*, BALB/c mice were inoculated intranasally with a sublethal dose of either WU2 WT or WU2Δ*tig*^kan^. The lack of TF resulted in reduced bacterial loads in both the nasopharynx and the lungs of mice, as compared to inoculation with WU2 WT (Fig. 6; n = 5 in each group, Student *t*-test, p = 0.0001 and p = 0.0031, respectively). These results confirm the involvement of TF in *S. pneumoniae* virulence. The growth rate and colony morphology of the WU2Δ*tig*^kan^ strain were similar to those of its parental WU2 WT strain (data not shown), as described for WU2Δ*tig*^Erm^. To reduce animal suffering we did not repeat the virulence test with the complemented strain.

## Discussion

The aim of this study was to determine the vaccine potential of TF and elucidate the involvement of TF in *S. pneumoniae* pathogenesis. Initially, we confirmed our previous findings that TF is localized to the CW of *S. pneumoniae*, although it does not have a signal peptide or any other known export sequences. TF lacks a human homologue and is conserved among all the clinical strains of *S. pneumoniae* tested, in addition to all of the sequenced pneumococcal strains available in the NCBI database. We show that mice immunized with rTF are protected against a lethal challenge by *S. pneumoniae*. We hypothesized that TF contributes to *S. pneumoniae* pathogenesis by facilitating bacterial adhesion to host cells. Indeed, we found that rTF and the anti-rTF antiserum inhibited *S. pneumoniae* adhesion to lung derived epithelial cells. Moreover, bacteria lacking TF showed reduced adhesion to cultured lung derived epithelial cells, as well as to neuronal and glial cells *in vitro*. A deletion of TF reduced virulence in a mouse model, as compared with WU2 WT *in vivo*. These findings indicate that TF may serve as a candidate vaccine whose coverage is much broader than that of the currently available pneumococcal vaccines.

Yang *et al*., (2005 and 2007) showed that the immunization of mice with *Brucella melitensis*-derived *tig* DNA or rTF elicited a protective immune response^40,41^. Similarly, the immunization of mice with *S. pyogenes*-derived rTF elicited a protective immune response against a lethal challenge^31,32^. Yet, the ability of a certain protein to elicit a protective immune response against a specific bacterium does not necessarily indicate an ability to elicit protection against another bacteria. For example, fructose bis phosphate aldolase (FBA) was found to be localized to the bacterial CW in both *S. pneumoniae* and *S. pyogenes*. However, albeit the high homology between the two proteins (79–89%), rFBA elicited a protective immune response against *S. pneumoniae* challenge but not against *S. pyogenes* challenges in mice immunized with the respectively derived rFBA^20,31^. Similarly, rDnaK from *S. pyogenes* elicited a protective immune response against a challenge in mice^31^, while the homologous protein, derived from *S. pneumoniae*, failed to protect mice from an *S. pneumoniae* challenge^20^. Therefore, the vaccine potential of a protein should be tested in the context of the relevant bacterium species.

DnaK is a chaperon involved in nascent protein folding^42^, which functions downstream of TF. The deletion of either *tig* or *dnaK* in *E. coli* did not affect either protein aggregation or growth phenotype under standard laboratory conditions^43–45^. However, the deletion of both *tig* and *dnaK* resulted in a strain that demonstraed increased protein aggregation and was lethal at temperatures higher than 30 °C^46,47^. These studies imply that the function of TF in protein folding can be replaced by that of DnaK^46^. Notably, DnaK is also localized to the CW of *S. pneumoniae, S. pyogenes, Clostridium thermocellum*, *Mycobacterium tuberculosis* and *Listeria monocytogenes*^20,27,31,48–50^.

CW localized TF may contribute indirectly to *S. pneumoniae* pathogenesis via the alteration in folding other proteins, thus affecting their function. Our result, that immunization with rTF protected mice from a *S. pneumoniae* challenge, albeit the presence of DnaK in the CW, which should have replaced the TF function in protein folding^51^, suggests that CW-associated TF is not involved in the folding of extracellular proteins but rather in bacterial pathogenicity.

TF in *E. coli* was demonstrated to affects the maturation, membrane binding and translocation through the membrane of pro-OmpA, in addition to being involved in the protection and folding of the nascent protein extruding from the ribosome^52^. The maturation of pro-OmpA is mediated, among others, by the peptidyl-prolyl isomerization domain of TF^52^. Streptococcal lipoprotein rotamase A (SlrA), which is a functional peptidyl-prolyl isomerase, was demonstrated to be indirectly involved in the adhesion of *S. pneumoniae* to the host^53^. Hermans *et al*.^53^, have shown that the adhesion and penetration of an *S. pneumoniae* D39Δ*slrA* mutant to A549 cells and to the human nasopharyngeal epithelial cell line Detroit 562 were significantly reduced compared to the parental D39 WT strain. However, neither the rSlrA protein nor an anti-rSlrA antiserum inhibited bacterial adhesion to the cells. Hence, the authors concluded that the involvement of SlrA in adhesion is indirect, possibly by affecting the structure/function of as yet unknown protein(s)^53^. In the current study, we show that an rTF and an anti-rTF antiserum inhibited the adhesion of *S. pneumoniae* to A549 cells, suggesting that TF directly contributes *S. pneumoniae* adhesion to the host. The fact that rTF can compete only with CW TF and that anti-rTF antibodies can react only with CW TF but cannot penetrate the cytoplasmic membrane and react with the cytoplasmic TF highlights the different roles that TF may plays in each of the cellular locations.

We also tested the extent of adhesion of *S. pneumoniae* to A549^21^, NSC 34^38^ and U251^39^ cells. A549 cells were found to retain the morphological, biochemical and immunological characteristics of human type II lung epithelial cells and are widely used as a model to study pneumococcal interaction with human cells^21,35–37^. NSC 34^38^ and U251^39^ cell-lines were used due to their neural origin. *S. pneumoniae*, *Neisseria meningitidis* and *Haemophilus influenzae* are the major bacterial pathogens causing meningitis, with *S. pneumoniae* being responsible for two thirds of meningitis cases in the developed world^54,55^. NSC 34 is a mouse neuroblastoma-spinal cord hybrid immortalized cell-line^38^, which has been used to study differentiation and characteristics of motor neuron^56,57^. The glioblastoma multiforme derived cell-line U251^39^ has been used to study the nature of glioblastoma multiforme and its susceptibility to anti-cancerous drugs^58,59^. To our knowledge, the current study is the first to use NCS 34 and U251 cell-lines as targets for *S. pneumoniae* adhesion. The deletion of *tig* significantly reduced bacterial adhesion to A549, NCS 34 and U251 cells, establishing that TF contributes to *S. pneumoniae* adhesion to various targets in the host. Moreover, the *tig* deletion significantly reduced bacterial loads in both the nasopharynx and the lungs of mice following a challenge, further validating the involvement of TF in *S. pneumoniae* pathogenesis. Importantly, the deletion of *tig* did not affect the bacterial growth rate at midlog, thus excluding the possibility that the observed differences in the extent of adhesion resulted from attenuated growth of the mutant. Similarly, the deletion of *tig* did not significantly affect the growth of *Bacillus subtilis*^60,61^, *Sinorhizobium meliloti*^62^, *L. monocytogenes*^63^, *S. mutans*^64,65^ or *S. pyogenes*^66^.

Taken together the ability of rTF to elicit a protective immune response against a lethal challenge, the contribution of TF to *S. pneumoniae* adhesion, the conservation of the protein among *S. pneumoniae* strains and the lack of a human homologue, all suggest that rTF can be considered as a candidate vaccine with a broad coverage.

## Methods

### Ethics statement

Experiments involving animals were carried out in strict accordance with the recommendations in the Guide for the Care and Use of Laboratory Animals of the National Institutes of Health. The protocol was approved by the Institutional Animal Care and Use Committee of the Ben-Gurion University of the Negev, Beer Sheva, Israel (Permit number: 53.08.08).

#### Mouse strains

Seven-week-old BALB/cOlaHsd (BALB/c) female mice (Harlan Laboratories, Israel) were used in this study. The mice were housed in sterile conditions under 12-h light/dark cycles and fed Purina Chow and tap water *ad libitum*.

#### Immunization of mice

BALB/c mice were immunized subcutaneously (SC) with 25 µg of rHis-TF emulsified with complete Freund’s adjuvant (CFA) and subsequently boosted (days 14 and 28) with 25 µg of rHis-TF emulsified in incomplete Freund’s adjuvant (IFA). Control mice were immunized with the adjuvant only.

#### Lethal challenge of mice

Following immunization on day 42, the mice were challenged intranasally, under deep anesthesia using isoflurane (Piramal Critical Care Inc., PA, USA), with a lethal dose (1 × 10^8^) of *S. pneumoniae* serotype 3 strain WU2^67^. Mice were humanely euthanized by CO_2_ asphyxiation, as recommended by the AVMA Guidelines for Euthanasia in Animals: 2013 Edition (https://www.avma.org/KB/Policies/Documents/euthanasia.pdf), if they became moribund or showed evidence of distress. The following criteria were considered sufficient evidence of distress to warrant such intervention and minimize pain and suffering to animals: severe weight loss (20% body weight); reluctance or inability to move freely; the appearance of bristle fur; social disengagement; refusal or inability to eat or drink. No analgesic treatment was provided as such treatment could alter the immune response and therfore independently affect the outcome of the experiments^68^. Morbidity and mortality were monitored daily.

#### Intranasal inoculation and bacterial load determination

Six to seven weeks old BALB/c mice were inoculated with a sublethal dose (5 × 10^7^) of either WU2 WT (n = 5 in each experiment) or WU2Δ*tig*^Kan^ (n = 5 in each experiment). Mice were humanely sacrificed 48 h later by CO_2_ asphyxiation. The nasopharynx and lungs were excised, homogenated and plated onto blood agar plates for enumeration. The results presented are a summary of three experiments performed on different occasions.

#### Preparation of rabbit and mouse anti-rTF antisera

Three-month-old white albino rabbits (Harlan Laboratories, Israel) or 6-week-old BALB/c mice were immunized SC with 200 µg or 25 µg rHis-TF, respectively, emulsified with CFA (1:1) in the first immunization or with IFA in booster immunizations in 2 week intervals. Two weeks after the final immunization, sera were obtained from blood collected from the rabbits’ marginal ear vein and by cardiac puncture following deep anesthesia from the mice.

### Reagents

Unless otherwise stated, all chemicals and biochemical materials were of the highest purity available and were purchased from Sigma-Aldrich (St. Louis, MS, USA).

### Bioinformatic analysis of trigger factor

“The amino acid sequence of Trigger Factor protein from *Streptococcus pneumoniae* serotype 4 (strain ATCC BAA-334/TIGR4) was retrieved from UniProt, ID Q97SG9 (TIG_STRPN). Orthologous proteins of Q97SG9 were retrieved from EggNOG (ID ENOG4105DEA), a database of orthologous groups and functional annotation, and filtered to only include proteins of the genus *Streptococcus* (n = 52). Amino acid sequences of Q97SG9 and all *Streptococcus* orthologs from EggNOG (n = 53) were multiply aligned using Muscle and visualized in Jalview (Supplementary Table S1 and Supplementary Fig. S1). The leading 33 amino acids of protein 888048.HMPREF8577_1918 (*S. parasanguinis* ATCC 903) were trimmed since they were not homologous to any of the other protein sequences. Below the multiple sequence alignment Jalview adds an automatically calculated conservation track, which is visualized as a histogram of the conservation score for each column. Conserved columns are indicated by ‘*’, and columns with mutations where all properties are conserved are marked with a ‘+’ (Supplementary Fig. S1)”.

B-cell epitope prediction analysis of Q97SG9 was conducted using four different web tools: 1. ABCpred Prediction Server (http://crdd.osdd.net/raghava/abcpred/), where epitopes with ranks 1–5 were retrieved; 2. LBtope: Linear B-cell Epitope Prediction Server (http://crdd.osdd.net/raghava/lbtope/), where epitopes with probabilities 61–100% were retrieved; 3. BCPreds: B-cell epitope prediction server (http://ailab.ist.psu.edu/bcpred/); and 4. Bepipred Linear Epitope Prediction 2.0 (http://tools.iedb.org/bcell/).

All programs were run using default parameters. Prediction was done using the linear amino acid sequence of Q97SG9, since (according to UniProt) solved structures of Q97SG9 are currently unavailable. The predicted epitopes from the four web tools were added as annotations below the conservation track in Jalview. In cases where epitopes were ranked by the prediction program, ranks were indicated in the display (with 1 designating the best predictions and higher numbers designating lower ranks); otherwise, the epitopes were indicated as 0.

### Bacterial strains and growth conditions

The *S. pneumoniae* encapsulated serotype 3 WU2^67^ strain and the unencapsulated serotype 2 D39-derived, R6 strain (ATCC, MD, USA) were used. Pneumococci were grown in Todd-Hewitt broth supplemented with 0.5% yeast extract (THY) and growth was monitored at O.D_620_ or on blood agar plates, as previously described^20^. In addition, 60 *S. pneumoniae* clinical strains were obtained as a courtesy of GSK, Belgium (Supplementary Table S2). Two *Escherichia coli* strains were used as cloning vectors: DH5α UltraMAX (DH5α; Invitrogen Corp, CA, USA) and BL21 (DE3) pLysS (BL21; Promega Corp, WI, USA) and were grown in Luria broth (LB) Lennox.

### Cloning, expression and purification of rTF

The nucleotide sequence of the locus SP_RS01985, coding for the WP_000116479.1 protein was amplified by PCR from the genomic DNA of WU2 pneumococcal strain, according to the NCBI published sequence of TIGR4 serotype 4 strain. PCR was performed with the coding region for TF (*tig*) and *tig* pET32+ forward and reverse primers (see Supplementary Table S3). The amplified product was digested with *Bam*HI and *Xho*I (Takara Biomedicals, Japan) and cloned into the pET32a+ expression vector (Novagen, China) prior or following the removal of the thioredoxin reductase (Trx) domain, flanked with *Nde*I using the *Nde*I restriction enzyme (Takara Biomedicals, Japan). pET32a+^*trx+tig*^ and pET32a+^*tig*^ were transformed into *E. coli* DH5α cells. Verification of sequence identity was performed by plasmid insert sequencing (data not shown) and found to be in frame. The vector was purified using the Qiagen High-Speed Plasmid Maxi Kit and transformed into *E. coli* BL21. Bacteria were grown overnight, and expression of rTF was induced with 0.5 mM isopropyl-β-d-thiogalactopyranoside (IPTG) for 5 h. The cells were harvested and lysed, and the protein was purified under native conditions and then dialyzed against PBS for imidazole removal. r-His-Trx-TF was used to test *S. pneumoniae* adhesion to cultured cells (as described below) and rHis-TF was used in immunization experiments. Both recombinant proteins were ~95% pure (data not shown). We also purified rHis-Trx from pET32a+ ^35,36^ to serve as a negative control for the adhesion experiments.

The untagged rTF protein was commercially produced by Protein laboratories, Ltd. (Israel), which amplified *tig* from TIGR4 genome^69^, and cloned, expressed and purified the protein as previously described^28,33^. Validation was also performed by the company using MS.

### Anti-rTF antibody titers in immunized mice

Microtiter plates (F96 Maxisorp Nunc, ThermoFisher) were coated with 1 µg/ml solution of rHis-TF in bicarbonate buffer pH 9.6. ELISA was performed as previously described^33^. Sera obtained from mice after the second (TF II) and third (TF III) rHis-TF immunizations served as primary antibodies. Serum obtained following the third immunization of mice with rHis-PtsA^28^ (PtsA III) served as a negative control.

### Immunoblot analysis

rHis-TF (1 µg) or CW protein fraction (30, 40, 45 or 60 μg) were separated by SDS-PAGE under reduced conditions and transferred to nitrocellulose membranes (Bio-Rad, CA, USA), as previously described^28^. Detection of TF was performed using rabbit anti-rHis-TF antiserum and peroxidase-conjugated AffiniPure F(ab9)_2_ fragment goat anti-rabbit IgG (H + L; Jackson Laboratories, ME, USA). Membranes were developed using MicroChemi 4.2 (DNR, Israel), and thereafter the molecular weight markers were added to the chemiluminescent image (Markers overlay). Either pre-immune serum or exclusion of the primary antibody were used as negative controls.

### Anti rTF monoclonal entibody (mAb) production

BALB/c mice were immunized 4 times, in 2 week intervals, with 25 µg rHis-TF in the presence of CFA in primary immunization and IFA in booster immunizations. Two weeks after the last immunization splenocytes were harvested for fusion with NSO cells by standard techniques. The hybridomas were tested for reactivity with rHis-TF by ELISA. Positive clones were further subcloned, and the clone with the highest reactivity to the protein was adapted to the serum-free media. The cells were expanded, and the mAb was purified from the culture supernatant by Protein G affinity chromatography. The reactivity of the purified mAb was reconfirmed by ELISA (data not shown) before sorting by FACS. The purified mAb preparations were in the range of 0.6–1.3 mg/mL^−1^. Purified IgG from naive mice was used as a control. An additional control was the exclusion of the primary antibody (data not shown).

### Flow cytometry of *S. pneumoniae*

Flow cytometry was performed as previously described^28^. Briefly, R6 bacteria were first stained with 5(6)-Carboxyfluorescein diacetate *N*-succinimidyl ester (CFSE), then washed, incubated with anti-rHis-TF mAb or isotype control mouse serum, or phosphate buffer seline (PBS), washed again, and stained with Alexa Fluor 647^®^-conjugated goat-anti-mouse-IgG (Jackson ImmunoResearch, PA, USA). Flow cytometry was performed using a FACSCalibur flow cytometer (Becton Dickinson (BD), CA, USA). CFSE staining was visualized by excitation with an argon laser (488 nm) and detection through FL1 (530/30 BP). Cells positive for CFSE were gated as live cells and evaluated for their 647 nm staining intensity (excitation by red diode laser 633/635 nm and emission detected through FL4 661/12 BP). The cytometer collected 30,000 events (FL1) and 10,000 CFSC positive by FL1 were gated and analyzed by FL4 at 647 nm. Software used for collection was CellQuest™ (BD and Company, NJ, USA). For analysis of the data, we used FlowJo software version 9.2 for Macintosh (Tree Star, Inc., Ashland, OR, USA).

### Cell lines

A549 cells (lung adenocarcinoma cells; ATCC, MD, USA)^21,35–37^ and NSC 34 cells (murine motor-neuron like cells)^38^ were grown in Dulbecco’s modified Eagle medium (DMEM; Biological Industries, Israel). U251 cells (human glioblastoma multiforme derived cells)^39^ were grown in RPMI 1640 (Biological Industries, Israel). All media were supplemented with 10% fetal calf serum, penicillin and streptomycin (100 µg/ml each) and cells grown at 37 °C in a humidified incubator.

### Evaluation TF contribution to pneumococci adhesion to cultured human cell-lines

A549 cells were cultured on 96-well plates, as previously described^25^. The extent of *S. pneumoniae* adhesion to the cells in the presence of r-His-Trx-TF (0–500 nM) was tested using MOI of ~100:1. rHis-Trx served as a negative control. The extent of *S. pneumoniae* adhesion to A549 cells in the presence of anti-rTF antiserum was tested as previously described^25,28^. Similar results were obtained with the His-rTF protein (data not shown) but, we present here only the results of rHis-Trx-TF, which provided us with an internal negative control as the expressed and purified rHis-rTrx protein that excluded the possibility that either rHis or rTrx are involved in adhesion. Evaluation of WU2 WT, WU2Δ*tig*^Erm^ mutant and WU2Δ*tig*^*tig*/Erm/Kan^ complemented strains adhesion was performed with A549^21^, U251 and NSC 34 cells. The results presented are the summation of data obtained from three independent experiments, each performed in triplicates.

### Preparation of WU2Δ*tig* mutant (kanamycin or erythromycin resistance)

To create null mutants of the *tig* gene, either the kanamycin (Kan) or erythromycin (Erm) resistance cassette was inserted into the coding sequence of *tig* via homologous recombination, as previously described^70^, with minor modifications. The upstream and downstream flanking regions of *tig* were amplified by PCR from the DNA of WU2 using primers designed according to the TIGR4 sequence. Primer combinations included upwing-F/upwing-R (see Supplementary Table S3, Upwing WU2Δ*tig*), for the upstream region of 800 bp and downwing-F/downwing-R, for the downstream region of 702 bp (Supplementary Table S3, Downwing WU2Δ*tig*). The Kan cassette was amplified by PCR from the genome of CP1250^71^ using the primer combination Kan AB-F/Kan AB-R (see Supplementary Table S3, Kan AB cassette). The Erm cassette was amplified by PCR from the genome of WU2Δ*nox*^*Erm*^ ^72^ using the primer combination Erm AM-F/Erm AM-R (see Supplementary Table S3, Erm AM cassette). The PCR products were digested with the corresponding restriction nucleases, as specified in Supplementary Table S3, purified, ligated and transformed into WU2 in the presence of the competence stimulating factor CSP1 and CaCl_2_. Transformants were selected on THY plates solidified with 1.5% agar, containing kanamycin (80 µg/ml) or erythromycin (125 µg/ml). Verification of *tig* deletion was done by PCR using specific primers (see Supplementary Table S2, Kan AB cassette for Kan resistance and Erm AM cassette for Erm resistance).

### *Cis*-complementation of WU2Δ*tig*^Erm^

To confirm that mutation of *tig* introduced no polar effects, WU2Δ*tig*^Erm^ was complemented with an intact copy of the gene using pCEP, which is a non-replicative plasmid that allows controlled gene expression under its native promoter, following ectopic integration into the chromosome^73,74^. Briefly, *tig* was amplified from WU2 with *tig* pCEP-F and *tig* pCEP-R primers (see Supplementary Table S3, *tig* pCEP complementation), which introduce *NcoI* and *PstI* sites. The amplicons were then ligated into *NcoI* and *PstI* digested vector. An aliquot of ligation mixture was transformed into Stellar™ competent cells (Clonetech, France), as described by the manufacturer. The transformants were selected for kanamycin resistance (500 μg/ml). Successful ligation was determined by colony PCR using Mal-F and pCEP-R primers (see Supplementary Table S3, *tig* pCEP verification), whose recognition sites are localized immediately upstream and downstream of the cloning site, respectively. These primers amplified a 263 bp product in an empty vector, while they produce a product of 1747 bp in the recombinant clones (additional 1484 bp represents the cloned fragment containing *tig* of 1284 bp). The recombinant plasmid was purified using a commercial kit (Qiagen) and an aliquot was transformed into WU2Δ*tig*^Erm^, as described previously^75^, to produce the complemented strain WU2Δ*tig*^*tig*/Erm/Kan^. The transformants were selected on blood agar plates supplemented with erythromycin (125 μg/ml) and kanamycin (500 μg/ml). Integration of *tig* into the genome was confirmed by PCR (see Supplementary Table S3, Kan resistance in pCEP verification).

### Preparation of *S. pneumoniae* CW protein fraction

The isolation of the CW proteins was performed as previously described^27,76^.

### Statistical analysis

Normal distribution and sufficient sample size of small sample sized data sets were verified using the Shapiro-Wilk test and post-hoc statistical power analysis, respectively, to justify the use of one-way ANOVA for parametric data, followed by the Dunnett test for multiple comparisons. A one-tailed Student’s *t*-test with Welch’s correction was used for bacterial load comparisons between the two groups. Data are reported as the mean ± SEM, unless stated otherwise. Spearman correlations were used to assess the significance of change in adhesion assays. Survival of *S. pneumoniae*-inoculated mice was determined using the Log-rank (Mantel-Cox) test. Differences were considered significant at p < 0.05. All statistical analyses were performed with the software package in GraphPad Prism version 7 (La Jolla, CA, USA).

## Supplementary information

Supplementary tables and figures
