# Supplementary material for: Streptococcus pneumoniae Cell Wall-Localized Trigger Factor Elicits a Protective Immune Response and Contributes to Bacterial Adhesion to the Host
Source: Sci Rep. 2019 Mar 12;9:4295. doi: 10.1038/s41598-019-40779-0 (PMC6414539; doi:10.1038/s41598-019-40779-0)
Supplement: Supplementary file 1 — Supplementary tables and figures [file 41598_2019_40779_MOESM1_ESM.pdf]

Streptococcus pneumoniae Cell Wall-Localized Trigger Factor Elicits a Protective Immune Response and Contributes to Bacterial Adhesion to the Host

Aviad Cohen[1], Shani Troib[1], Shahar Dotan[2], Hastyar Najmuldeen[3,4], Hasan Yesilkaya[3], Tatyana Kushnir[1], Marilou Shagan[1], Maxim Portnoi[1], Hannie Nachmani[1], Rachel Benisty[1], Michael Tal[2], Ronald Ellis[2], Vered Chalifa-Caspi[5] Ron Dagan[6], Yaffa Mizrachi Nebenzahl[1]

Supplementary Table S1. Streptococcus orthologues of *S. pneumoniae* TF protein according to EggNOG**.**

Organism	Protein Identifier
Streptococcus pseudoporcinus SPIN 20026	910313.HMPREF9320_0768
Streptococcus sp. M143	563037.HMPREF0850_01437
Streptococcus anginosus F0211	706437.HMPREF0813_00040
Streptococcus pneumoniae R6	171101.spr0362
Streptococcus pyogenes M1 GAS	160490.SPy_1896
Streptococcus gordonii str. Challis substr. CH1	467705.SGO_0412
Streptococcus vestibularis F0396	904306.HMPREF9192_0259
Streptococcus macacae NCTC 11558	764298.STRMA_1793
Streptococcus mitis B6	365659.smi_1739
Streptococcus mitis bv. 2 str. SK95	1000588.HMPREF9965_0617
Streptococcus criceti HS-6	873449.STRCR_0597
Streptococcus infantis SK970	1035189.HMPREF9954_1690
Streptococcus sanguinis ATCC 49296	888049.HMPREF8578_0868
Streptococcus urinalis 2285-97	764291.STRUR_1838
Streptococcus sp. C150	435842.HMPREF0848_01467
Streptococcus cristatus ATCC 51100	889201.HMPREF9422_1782 , 889201. HMPREF9960_0869
Streptococcus porcinus str. Jelinkova 176	873448.STRPO_1764
Streptococcus sp. M334	563038.HMPREF0851_00868
Streptococcus downei F0415	904293.HMPREF9176_0139
Streptococcus infantis SK1302	871237.SIN_1169
Streptococcus australis ATCC 700641	888833.HMPREF9421_0069 , 888833. HMPREF9961_1100
Streptococcus suis 05ZYH33	391295.SSU05_0328
Streptococcus sp. oral taxon 056 str. F0418	904294.HMPREF9182_1707
Streptococcus mitis NCTC 12261	246201.SM12261_1026
Streptococcus agalactiae NEM316	211110.gbs0104
Streptococcus mutans UA159	210007.SMU_91
Streptococcus sp. oral taxon 071 str. 73H25AP	864570.HMPREF9189_1381
Streptococcus infantarius subsp. infantarius CJ18	1069533.Sinf_0279
Streptococcus equinus ATCC 9812	525379.HMPREF0819_1699
Streptococcus dysgalactiae subsp.	663952.SDD27957_01545

dysgalactiae ATCC 27957	
Streptococcus uberis 0140J	218495.SUB0323
Streptococcus parasanguinis ATCC 903	888048.HMPREF8577_1918
Streptococcus sanguinis SK36	388919.SSA_1998
Streptococcus ictaluri 707-05	764299.STRIC_1961
Streptococcus oralis Uo5	927666.SOR_1623
Streptococcus peroris ATCC 700780	888746.HMPREF9180_0373
Streptococcus salivarius 57.I	1046629.Ssal_02041
Streptococcus parauberis NCFD 2020	873447.SPB_1630
Streptococcus pseudopneumoniae IS7493	1054460.SPPN_02555
Streptococcus infantis SK1076	1005705.HMPREF9967_1624
Streptococcus mitis SK597	585204.SMSK597_0283
Streptococcus pneumoniae D39	373153.SPD_0365
Streptococcus mitis bv. 2 str. F0392	768726.HMPREF9178_0910
Streptococcus thermophilus LMG 18311	264199.stu0132
Streptococcus infantis X	997830.HMPREF1124_0801
Streptococcus anginosus SK52 = DSM 20563	1000570.HMPREF9966_1814
Streptococcus macedonicus ACA-DC 198	1116231.SMA_0290
Streptococcus mitis ATCC 6249	864567.HMPREF8571_0479
Streptococcus equi subsp. zooepidemicus	40041.SZO_02510
Streptococcus constellatus subsp. pharyngis SK1060 = CCUG 46377	1035184.HMPREF1042_1849
Streptococcus oralis ATCC 35037	655813.SMSK23_0907
Streptococcus mitis SK321	585202.SMSK321_0001

Supplementary Table S2: List of *Streptococcus pneumoniae* clinical strain used in the current study

Strain code	Strain Name	TF
GSK1	SPn 9 KOL 112 Pol 161/95 BAB 2	+
GSK2	SPn 9 KOL 136 Pol 187/95 BAB 4	+
GSK3	SPn 9 KOL 136 Pol 187/95 HoeP 4	+
GSK4	SPn 9 KOL 270 Pol 206/95 BAB 5	+
GSK5	SPn 9 KOL 283 Pol 221/95 HoeP 5	+
GSK6	SPn 9 KOL 462 Pol 311/95 BAB 7	+
GSK7	SPn 9 KOL 578 Pol 373/95 2	+
GSK8	SPn 9 KOL 113 Pol 162/95 BAB 1	+
GSK9	SPn KOL 113 Pol 162/95 HoeP 3	+
GSK10	Pn 9V Lot 1	+
GSK11	SPn 9V 112 161/95	+
GSK12	SPn 9V 112 161/95 14	+
GSK13	SPn 9V 113 162/95	+
GSK14	SPn 9V 136 187/95	+
GSK15	SPn 9V 270 206/95	+
GSK16	SPn 9V 283221/95	+
GSK17	SPn 9V 578 373/95	+
GSK18	SPn 9V 9220/38 2	+
GSK19	SPn 7F OSLO,GrO134 10	+
GSK20	SPn 7F Denmark 332/95 10	+
GSK21	SPn 7F VERBIST 149 10	+
GSK22	SPn 7F Denmark 201/95 9	+
GSK23	SPn 7F Lyon 95009 151 8	+
GSK24	SPn 7F Denmark 212/95 6	+
GSK25	Pnf N 1/1	+
GSK26	SPn 1/37 (140278) 9	+
GSK27	SPn 3 Holland 1656	+
GSK28	SPn 3/43 5	+
GSK29	SPn 4/2656 13	+
GSK30	Pn 4/2723	+
GSK31	Pn 5 Ambrose	+
GSK32	SPn 5 Ambrose 14	+
GSK33	Pn 5/94	+
GSK34	SPn 6A 6/2709	+
GSK35	9/27 11	+
GSK36	Pn 9/72	+
GSK37	Pn 10/10A	+
GSK38	SPn 14 2641/39 16	+
GSK39	Pn 14/58	+
GSK40	14/ATCC-3 G314 Lot 1	+
GSK41	SPn 14/88	+
GSK42	Pn 15/15F1 (79)	+
GSK43	Pn 15B/3449/39	+
GSK44	Pn 15/6315 Lot. 1 ATCC	+
GSK45	17/17F	+
GSK46	Pn 17/17F Rose	+
GSK47	Pn 18/18C	+
GSK48	SPn 18/18C 10	+
GSK49	SPn 19/19F 2737 20	+
GSK50	SPn 19/19F 18	+
GSK51	Pn 19/81	+
GSK52	SPn 19/2737 Is.(D.D.)	+
GSK53	SPn 23F 14	+
GSK54	Pn 24/24F	+
GSK55	Pn 25/67 Lot 1	+
GSK56	SPn 33b	+
GSK57	SPn 33C	+
GSK58	Pn 33/334	+
GSK59	Pn 35B	+
GSK60	Pn 35/35F	+
GSK61	S.Pn. 8/8 L	+
GSK62	S.Pn. 3/43	+
GSK63	S.Pn. 4/2656	+
GSK64	S.Pn. 25/25L	+
GSK65	S.P.n 12/12F	+
GSK66	S.Pn. 18/18C	+
GSK67	S.Pn. 14/88	+
GSK68	N.M. 3/43	+
GSK69	S.Pn. 14/58	+
GSK70	S.Pn. 1/37	+

Supplementary Table S3: Primers used in the current study

Primer	Restriction site	Sequence
tig pET32+ expression	*Bam*HI *Xho*I	F 5'-CG**GGATCC**ATGTCTGTATCATTTGAAAAC-3'<br>R 5'-CC**CTCGAG**TTATTTTACTGTTGCTGTGC-3'
Upwing WU2Δ*tig*	*Xho*I	F 5'-AGTGGACGAGGTTAGTCGCGT-3'<br>R 5'-TATA**CTCGAG**TGACTGTCCTGATCGATTTGTCCTG-3'
Downwing WU2Δ*tig*	*Bam*HI	F 5'-AATCG**GGATCC**CCTGAATTGGTGGGTTTTCTGATGC-3'<br>R 5'-GTACCGAGGGACGGCAGGGAT-3
Kan AB cassette	*Xho*I BamHI	F 5'-GCGC**CTCGAG**CCGTTTGATTTTTAATGGATA-3'<br>R 5'-AATCGGGATCCCTAGGTACTAAAACA-3'
Erm AM cassette	BamHI ApaI	F 5'-CCGC**GGATCC**AGTCGGCAGCGACT-3'<br>R 5'-CC**GGGCCC**AAAATTTGTTTGAT-3'
tig pCEP complementation	NcoI PstI	F 5'-**CCATGG**AGTACCTATCGTGATTGGAG-3'<br>R 5'- **CTGCAG**TCAGTTCTTGCCATC-3'
tig pCEP verification	Mal pCEP	F 5'- GCTTGAAAAGGAGTATACTT -3'<br>R 5'- AGGAGACATTCCTTCCGTATC -3'
Kan resistance in pCEP verification		F 5'- GAGGTGCTACCATGGCGCGCA -3'<br>R 5'- CTAAAACAATTCATCCAGTAA -3

Supplementary Fig. S1. MSA with a B-cell epitope prediction

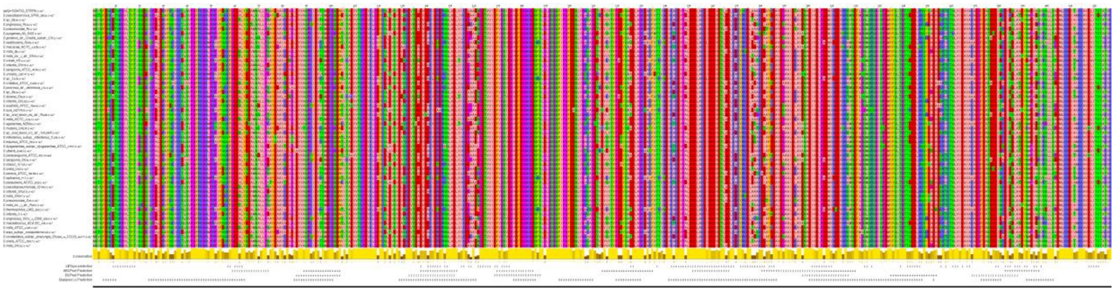

Multiple sequence alignment of *S. pneumoniae* TF protein and its Streptococcus orthologues annotated by conservation score and predicted B-cell linear epitopes. Alignment was performed with Muscle and conservation score was calculated by Jalview. B-cell epitope predictions of *S. pneumonia* TF amino acid sequence with LBtope, ABCpred, BCPreds and Bepipred are indicated by their prediction rank (with 1 designating the highest rank) or by zeros, if ranks are not provided.

Supplementary Fig. S2. Cell wall localization of TF

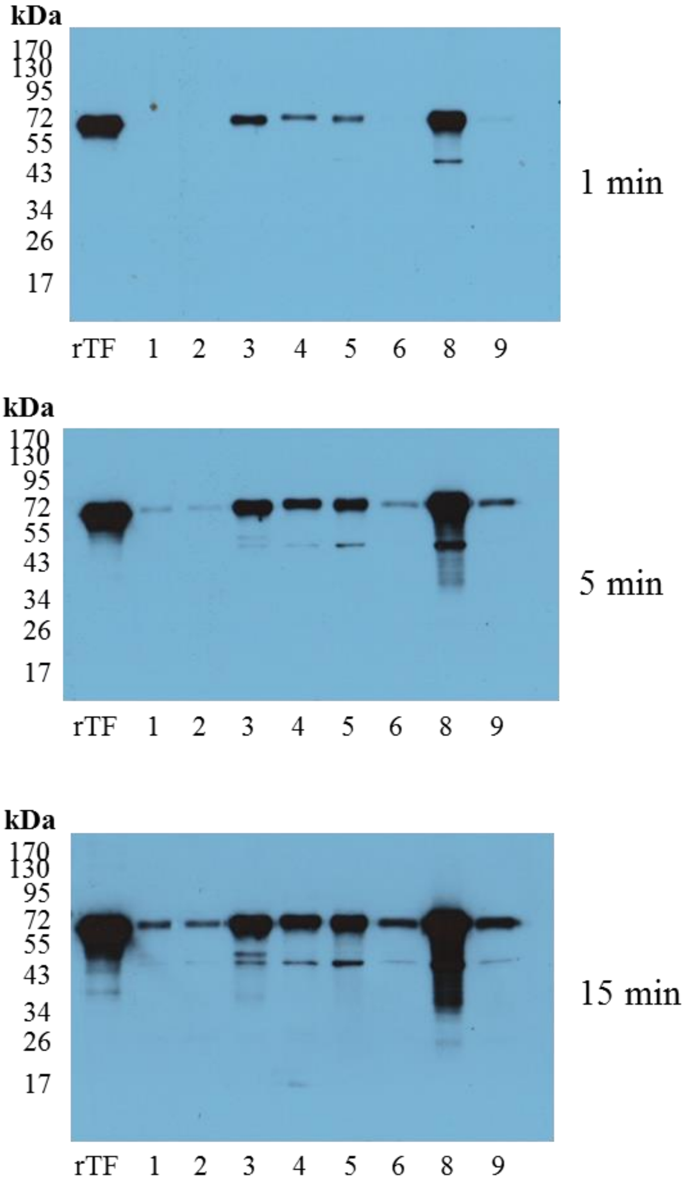

Thirty micrograms of protein of CW fractions from 60 *S. pneumoniae* clinical strains (see Supplementary Table S1, online) were loaded per lane, subjected to SDS-PAGE, and immunoblotted. Purified untagged rTF (0.01 μg) was loaded as a positive control. A representative full length blots are shown following exposure for 1, 5 and 15 min. The blot representing 15 min. exposure is cropped and show in Fig. 2b

Supplementary Fig. S3. TF immunogenicity

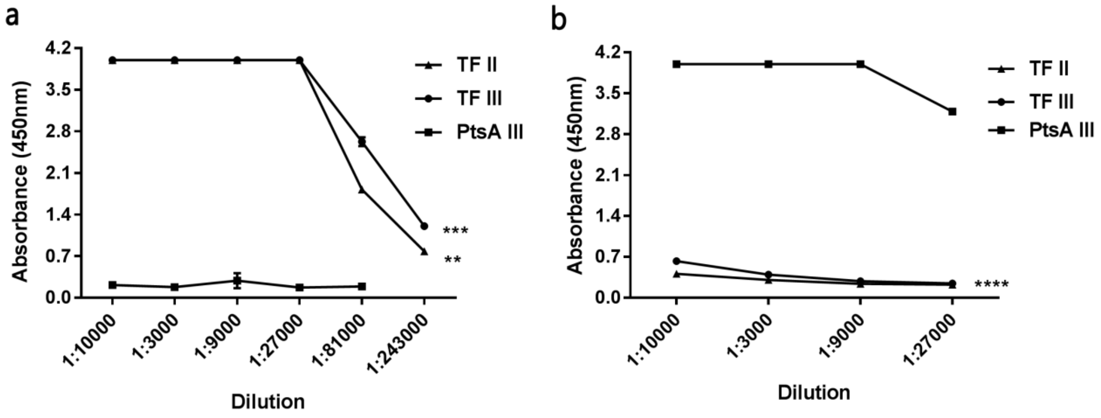

Microtiter plates were coated with 1 µg/ml of rTF (A) or rPtsA (B). Sera obtained from mice following the second and the third immunizations with rTF (TF II and TF III, respectively) or following the third immunization with rPtsA (PtsA III) were serially diluted and used as the primary antibodies. a. The antibody response following TF II and TF III was compared to that of an anti-rPtsA antiserum (one-way ANOVA with the Dunnett post-hoc test, **p = 0.0014, ***p = 0.0008). b. Reaction of anti-rTF antisera tested on rPtsA-coated plates, in comparison to anti-rPtsA antiserum (one-way ANOVA with the Dunnett post-hoc test, ****p = 0.0001).

Supplementary Fig. S4. Growth curves of the WU2 WT, WU2Δ*tig*[Erm], and U2Δ*tig*[*tig*/Erm/Kan] strains

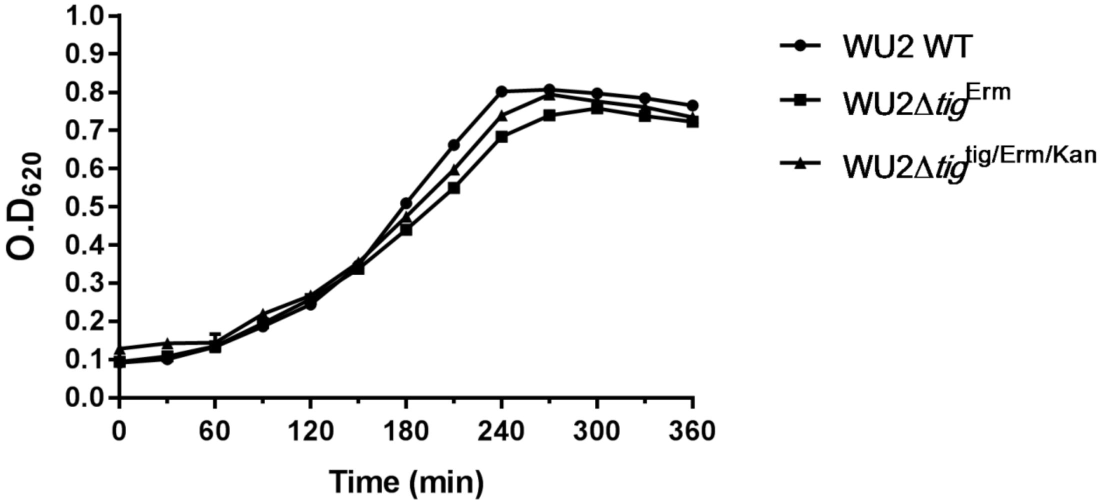

Pneumococci were grown in a Todd-Hewitt broth supplemented with 0.5% yeast extract (THY) and $O.D_{620}$ was measured.
